# Dietary Factors Influencing the Caries Status of Adults in Karachi, Pakistan: Initial Findings

**DOI:** 10.3390/ijerph19126980

**Published:** 2022-06-07

**Authors:** Ambrina Qureshi, Nilofer F. Safdar, Hina Qureshi, Yasser F. AlFawaz, Khold Al Ahdal, Sara Shabib, Khulud A. Al-Aali, Mustafa Naseem, Fahim Vohra, Tariq Abduljabbar

**Affiliations:** 1 Department of Community and Preventive Dentistry, Dow University of Health Sciences, Karachi 74200, Pakistan; 2 Nutritional Sciences, School of Public Health, Dow University of Health Sciences, Karachi 74200, Pakistan; nilofer.fatimi@duhs.edu.pk; 3 Department of Pathology, The Kidney Center Postgraduate Institute, Karachi 74200, Pakistan; dr.hinaqureshi02@gmail.com; 4 Department of Restorative Dental Sciences, College of Dentistry, King Saud University, Riyadh 11545, Saudi Arabia; yalfawaz@ksu.edu.sa (Y.F.A.); kalahdal@ksu.edu.sa (K.A.A.); sashabib@ksu.edu.sa (S.S.); 5 Department of Clinical Dental Sciences, College of Dentistry, Princess Nourah Bint Abdulrahman University, Riyadh 11545, Saudi Arabia; kaalaali@pnu.edu.sa; 6 Department of Community and Preventive Dentistry, Dow International Dental College (DIDC), Karachi 74200, Pakistan; mustafa.naseem@duhs.edu.pk; 7 Department of Prosthetic Dental Sciences, College of Dentistry, King Saud University, Riyadh 11545, Saudi Arabia; fvohra@ksu.edu.sa

**Keywords:** dietary factors, dental caries, sugar, cereals, Pakistan

## Abstract

Objective: The objective was to identify the relationship between the dietary factors related to increases in the number of dental caries among an adult population group. Methods: A cross-sectional study was conducted involving adult patients and their accompanying person, aged 18 years and above (*n* = 1730) visiting the dental outpatient department (OPD) of a public sector tertiary healthcare institute in Karachi, Pakistan. A 39-item Food Frequency Questionnaire (FFQ) was administered to the patients, followed by a dental caries assessment using Radke’s WHO criteria. Caries assessment data were transformed into the DMFT Index (D = decayed, M = missing, F = filled teeth). Factor analysis (FA) was performed using Stata v. 11.0, followed by assessing the internal consistency of the FFQ. Multilogistic analysis was performed to explore the association between dental caries (cut-off = 5) and other independent variables, considering a *p*-value < 0.05 as significant. Results: The mean age of participants in the group was 32.65 ± 10.49 years. The number of female participants (934; 54%) was higher than male participants (796; 46%). Of the total, 951 (52%) participants were married. The internal consistency value for the FFQ, as measured by Cronbach’s alpha, was 0.80. In addition to age and gender, out of four dietary patterns, only “cereals” were found to be significantly (*p* < 0.05) associated with increased DMFT. Conclusion: This study established that the high intake of cereals is a contributory factor to the high prevalence of dental caries among adults.

## 1. Introduction

Dental caries are defined as “a biofilm-mediated, diet-modulated, multifactorial, non-communicable, dynamic disease resulting in net mineral loss of dental hard tissues” [1]. Despite dental caries being a biofilm and dietary-dependent multifactorial disease, caries prevention is usually focused on fluoride exposure [2]. Sheiham and James [3] suggest that sucrose should be considered the sole substrate required for cariogenic oral bacteria to flourish and generate enamel-demineralizing acids, and they have criticized the use of the term “multifactorial” in the etiology of dental caries [3]. Furthermore, it is reported that managing fluoride levels and dental hygiene, along with social, behavioral, and biological factors, is of secondary importance in caries control if the control of sugar intake is accomplished [4]. 

A recent market survey conducted in Pakistan showed that 15% of the participants used dentifrices that were claimed by the manufacturers to contain the optimal level of fluoride, yet more than 90% of these users were suffering from active carious lesions [5]. Moreover, 84% of the water sources used in Pakistan are considered to have the safe levels of fluoride (1.5 mg/L) required to have positive health impacts [6]; however, 84% of the population still suffer from dental caries, irrespective of the fluoride content of the toothpaste they use [5]. This suggests that the available levels of fluoride in these toothpastes are questionable, and therefore, the dietary factors related to dental caries in developing countries such as Pakistan need to be redefined. 

Although the role of sugar as a causative factor for dental caries is unquestionable [7], the relationship between the intake of sugary foods and other components of food has not been completely determined [7,8]. In addition, other dietary components may or may not include carbohydrates. In Pakistan, the last national oral health survey was conducted almost two decades ago [9]. It is pertinent to mention that this national oral health survey did not take the dietary intake of the population into account, although the increase in caries in adults was attributed to aging and sugar intake [9]. Since then, there have been very few reports wherein specific dietary items have been correlated with dental caries in Pakistan, and those reports which have addressed this issue have done so only among children [10,11]. Hence, dietary patterns concerning dental caries are not well-defined in this population [9]. Moreover, increased weight or BMI is ubiquitous and, like the increase in dental caries, this has also been attributed to increased sugar intake. [12]. Therefore, it is critical to assess the relationship between dietary patterns and dental caries as a confounding or mediating factor in our study population [13].

Furthermore, several short diet surveys using the Food Frequency Questionnaire (FFQ) have been carried out around the globe, mostly among children, in an attempt to differentiate between children with and without severe early childhood caries (ECC) that are based on their diet [14,15,16,17]. Very few studies have considered the adult population [18,19], and one example of such a survey in the Asian adult population comes from Japan [20], where the dietary pattern is different from that in most of the other parts of Asia, particularly South East Asia. Considering the absence of data on the dietary factors related to dental caries in developing Asian countries, such as Pakistan, it is clear that this needs to be further explored. Based on these suggestions, it is critical to identify the pertinent dietary items or groups of items that define dietary patterns in our population. Therefore, the present study was conducted within a cohort of the adult population in Karachi, Pakistan to identify the dietary factors related to the increasing number of dental caries.

## 2. Materials and Methods

### 2.1. Ethical Statement

The study protocol was developed in line with the ethical principles of the declaration of Helsinki (amended in 2013). The Institutional Review Board revised and approved the research protocol of the study CSPRC-017/2021. The study was in agreement with the strengthening of the reporting of observational studies in epidemiology (STROBE) statement for reporting cross-sectional surveys. The period of the study was 3 months, from April 2019 to July 2019.

### 2.2. Study Settings and Participants

A cross-sectional study was conducted that included registered adult caries patients aged >18 years visiting the dental out-patient department of the Dow University of Health Sciences (DUHS). A convenience sampling method was adopted. The location was selected as it is a tertiary care setting in the public sector, with high patient numbers and accessible dental services, both in terms of service availability and cost-effectiveness. A minimum sample of 1730 was calculated, with a 95% confidence interval, a 5% level of significance, and a 5% margin of error. The sample size was increased to 1800 by adding an additional 5% of participants to overcome the loss of participants on follow-up. The sample size was calculated via the source available at https://www.surveysystem.com/sscalc.htm (accessed on 1 August 2021). Excluded from the study were subjects who had lost at least 25% of all teeth due to non-carious consequences (such as periodontal problems or orthodontic factors), participants with poor oral hygiene (plaque scores ≥ 2), those who were mentally or physically handicapped, and the frail and elderly. Excluded also were subjects who were suffering from any debilitating health condition (e.g., cancer, AIDS, bleeding disorders), patients having any form of dietary intolerance (such as lactose intolerance), non-consenting subjects, or participants having any missing data pertinent to the main study variables. Medical histories were collected by a clinical pathologist to rule out the participants as per the selection and exclusion criteria.

### 2.3. Study Questionnaire

After acquiring their medical histories, the participants were required to respond to a structured questionnaire. A single, trained interviewer (AQ) administered a structured, 3-part, pro forma, data questionnaire. The first part was used to record details such as age, gender, height (in meters (m)), weight (in kilograms (kg)) to calculate body mass index (kg/m^2^), frequency of brushing, number of dental visits, and marital status [21]. The name of the toothpaste and its fluoride concentration as claimed by the manufacturer (0 = less than 1100 parts per million and 1 = equal to or more than 1100 ppm) were recorded. Body mass index (BMI) was identified (using weight and height) and tabulated as categorical data based on the BMI categories for adults [21].

### 2.4. Food Frequency Questionnaire (FFQ)

The second part of this pro forma questionnaire included a 39-item FFQ that has been previously used and validated in the Japanese population [19]. The researchers claimed that it may be administered in any population group with any cultural background. This questionnaire was available in English and Japanese; however, we used the English version and translated it into Urdu (the local Pakistani language). The internal consistency of the questionnaire was assessed by Cronbach’s alpha (0.80). We did not change the response criteria, which remained the same based on participants recalling how often, on average, they consumed a given caries-related food during the past month (7 points, 0–6, categories ranging from “never” to “4 or more times per day”). We excluded six items from the original FFQ: lactic acid drink, jello, rice crackers, buns with bean jam filling, rice cakes, and bars of sweet, jellied adzuki bean paste because these items are not typically consumed by the Pakistani population. To increase the content validity, we added to the questionnaire two sweet items commonly consumed in Pakistan: “mithai” (similar to gulab jaman, jalebi, amrati) and “sweet deserts” (such as kheer, zarda, halwa). The questionnaire was pretested for input on content, language clarity, and layout on 25 subjects within a 2 week interval. All food item frequencies were entered as ordinal data.

### 2.5. Dental Caries Examination

The third part of this pro forma questionnaire contained the dental caries status of the participants, as determined by examination by a single, trained dental professional. Reference standard measurements were compared between the same examiner (Cronbach’s α = 0.80). The caries assessment was performed using Radike’s (WHO) caries scoring criteria. The DMFT index (decayed, missed, and filled) was used, and WHO criteria were used to examine this index. The total DMFT was generated by adding up the number of all decayed, filled, and missing teeth for each study participant, and subsequently, a 2-group cut-off was generated to be stored as a categorical variable (DMFT < 5 = 0; DMFT ≥ 5 = 1). Cavitated tooth decay on clinical examination was considered as a threshold for caries lesion detection to record the D component of DMFT using a basic examination kit, i.e., a mirror and probe under artificial light [1]. Non-cavitated or white spot lesions were not considered carious. This component of the examination was performed to avoid any confusion between white spot lesions and enamel hypoplasia/fluorosis, as suggested by the consensus group [1].

### 2.6. Statistical Analysis

All statistical analysis was performed on SPSS version 22 (SPSS Inc., Chicago, IL, USA). Continuous data included age, height, weight, and number of children of the participants. Factor analysis (FA) was performed after examining the correlation matrix through the polychoric correlation test (*p* < 0.05) and Kaiser–Meyer–Olkin (KMO) test of sample adequacy. KMO value of >0.7 was considered sufficient for the sample size selected (*n* = 1730). Using the prior criterion, it was decided to extract 4 factors through principal component analysis (PCA). The varimax method was used for factor rotation and a criterion of 0.55 was used for significant factor loading according to the sample size [14]. Three cut-off groups (tertiles) for each factor were generated. Internal consistency of FFQ using the Cronbach’s alpha (0.80) test was also performed using responses from the same subjects.

Descriptive analysis was performed using frequency percentages of the categorical variables and the mean (standard deviation) of the continuous variables. Logistic regression was performed to calculate crude odds ratios between DMFT (<5 = 0; ≥5 = 1) and age, gender, marital status, number of children, frequency of brushing, number of dental visits, fluoride level in toothpaste as claimed by the manufacturer, and tertiles of dietary factors. All independent variables with a *p*-value < 0.1 were incorporated into the multivariate model to calculate the adjusted odds ratio. As the level of significance was set at 5%, variables with a *p*-value ≤ 0.05 after the adjustment were maintained in the model.

## 3. Results

Table 1 presents a detailed description of the study participants. The mean age of participants in the group was 32.65 ± 10.49 years. The number of female participants (934; 54%) was more than male (796; 46%). Of the total, 951 (52%) participants were married and 779 (45%) were not married. When questioned about the frequency of brushing, 495 (28%) participants claimed they brushed once daily, whereas 1165 (67%) suggested that they brushed twice daily. When questioned about the number of dental visits, 240 (13%) participants alleged that they visit dentists every 6 months, whereas, 1389 (80%) reported that they visited dentists annually.

BMI was measured in kg/m^2^. The highest BMI (916; 53%) was measured in the age range of 18 to 22.9 years (916 participants; 53%), whereas the lowest BMI was found in the age group of ≥25 years (87 participants; 5%). There were 1232 (54%) participants who claimed that they used toothpaste that had fluoride levels of ≥1100 ppm. Mean DMFT scores were found to be 5.74 ± 1.86. Out of all the participants, 48.45% had a DMFT score of 4.

The final factor analysis was performed on 1730 participants. Items with a 100% “never” response were excluded from the factor analysis, which included “soda (not diet)”, “cocoa”, “breath mint”, “cough drops” and “gum (not sugar-free)”. “Sugared cereal” and “sugar in cereal” are considered the same by local people; therefore, we combined the two items as “sugared cereal”. “Canned fruit” was understood by the participants as fresh fruit with sugar added, so we replaced “canned fruit” with “fresh fruit with sugar”. We combined all kinds of candies (hard candy, sticky candy) as a single item due to these items being considered the same by the Pakistani population. Similarly, “donuts and muffins” and “cakes and pies” were combined as “cake, donuts, pie”. More than 50% of the data for “sweet dishes” was missing, so we had to exclude this item from the correlation matrix as per the exclusion criteria. The overall significance of the correlation matrix was thus based on a total of 24 items with Bartlett’s test of sphericity <0.001 (LR Chi2 = 590.54). KMO measure of sampling adequacy was 71.4%. The lowest eigen value calculated, that is, for the fourth factor extracted, was 1.34.

Table 2 presents loaded factors considering factor loading equaling 0.55 and the suggested labels. Only two items under each labeled factor had loading >0.55. Internal consistency of the FFQ was 81.79% (Cronbach’s alpha = 0.80).

**Figure 1:**
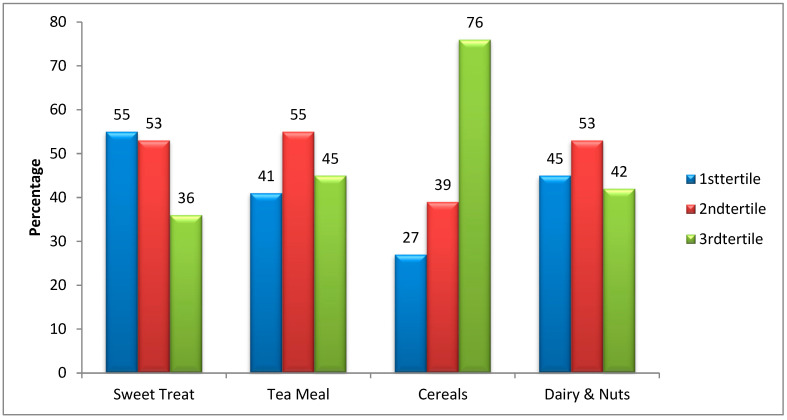
Association between DMFT and four dietary patterns (sweet treat, tea meal, cereals, dairy and nuts).

Figure 1 displays cluster bars for DMFT scores (as dependent variables) and “sweet treat” (A), “tea meal” (B), “cereal” (C), and “dairy and nut” (D) as independent variables. The highest tertile of intake within each food pattern consisted of 36% sweet treats, 42% for dairy and nuts, 45% for the tea-meal pattern, and 76% for cereals.

The crude and adjusted odds ratios for predictors, predicting dental caries ≥5 DMFT are presented in Table 3. Crude analysis showed that participants’ age, female gender, marital status, and 3rd tertile of the “cereal” dietary factor were found to have a significant association with DMFT ≥5, as compared to DMFT <5. After adjusting for significant variables, only the 3rd tertile of the “cereal” dietary factor remained as a significant predictor, as four times (OR = 4.36, *p* < 0.05) more likely to be associated with DMFT ≥5 as compared to DMFT <5. This variable was found to fit in the model with LR chi^2^ = 23.55 (*p* = 0.0003).

## 4. Discussion

This study presents the specific dietary patterns of the Pakistani adult population as related to their dental caries status. The study showed that the mean DMFT of all participants is reported to be >5. In that context, four distinct groups of dietary items (dietary patterns) were identified, with a total variance of approximately 75% of the variance in the participants’ food intake in this study. Although the dietary patterns were found equally distributed among the entire study population, irrespective of their caries severity, cereals were found to be significantly associated with the severity of caries, even after controlling for all other significant factors.

This study revealed that adults with higher intakes of cereals had five or more teeth with dental caries. On the contrary, cereals are considered a healthy food item among Sri Lankan adults, and those who consumed cereals were considered to have reduced caries levels as compared to those who consumed less cereals [22]. The expert panel members of the World Health Organization have affirmed that the increase in the risk of dental caries is associated with a frequent and total intake of simple sugars [23]. Since the food pattern “sugared cereals” in the current study consisted of sugar as well as plain cereals, this may accounted for the association between cereals and dental caries. It is also worth noting that only the highest (3rd) tertile for cereal intake was found to be associated with an increased level of dental caries as compared to the 1st or 2nd tertiles for cereal intake. Although cereals are usually considered readily available healthy breakfast items, many of these commercially available processed cereals are a source of high sugar [24]. A research team in Germany found that almost one-third of the commercially available cereals contain added sugars [25]. Therefore, consumers must check the ingredients and choose the cereals which are more wholesome and contain fiber and dried fruits [26].

In addition to this food pattern, the participants’ age, gender, and marital status were also found to have a significant association with dental caries. Females, with increasing age and married marital status, were found to have higher DMFT scores. Higher DMFT score with increasing age is an established factor [27]. The finding that the DMFT increased with age may not be surprising because caries are cumulative and chronic, and the DMFT measures past and present caries experiences [28]. Similarly, in the present study, women were found to be more commonly affected by dental caries as compared to men. A meta-analysis by Luckas and Largaespada stated the high caries rate in females can be attributed to the following three factors: early eruption of teeth, pregnancy, and frequent snaking patterns with easier access to food supply [29]. Moreover, hormonal fluctuation in women during menstruation and puberty are overall impacts on the buffering capacity of saliva, making the oral cavity more cariogenic compared to that of males [29]. It is pertinent to mention that both age and gender are the determinants of the dental caries that are mostly sugar-mediated [2,3]. In the current study, it was unusual to note that BMI was not related to dental caries, unlike other studies conducted among the Australian and Egyptian adult populations [30,31]. To date, no study has been conducted among the Pakistani adult population to look into the relationship between caries and BMI. However, recent work by Anzar et al., conducted among children, showed that reduced BMI and dental caries are associated [21]. Increased sugar intake has systematically been analyzed and been found to have an association with increased BMI [12], which in turn is an indicator of low socioeconomic status (SES) [32,33]. Although we did not record the SES of the study participants independently, and this was one of the limitations of the present study, it may be considered that the participants belonged to low-to-middle SES since more than half of the participants had normal BMIs [32,33]. This finding may explain the reason that BMI in this study was not associated with dental caries. Further studies may be conducted with larger population groups with equal representation from sub-samples of different ranges of BMI to explore any relationship between being underweight and dental caries or obesity and dental caries in adults.

It is worth noting that the other two food patterns, namely “sweet treats” and “tea meals” were not found to be associated with higher levels of dental caries, or to be more specific, with DMFTs greater than five. This may be because the intake of the food items in these two patterns was much lower in the 3rd tertile as compared to the items included in “cereals”. These factors may be explored further with a larger study sample since this preliminary stage of the study consists of a smaller sample size; it is difficult to explain this result in our population. However, the reason may be that almost three-quarters of the study participants used toothpaste that have fluoride levels equal to or greater than 1100 ppm. It is known that fluoride alters or delays the dose–response relationship between cariogenic food and dental decay [3,34].

One may question why the investigators set the DMFT cut-off at 5. Oral health researchers have confirmed that dental caries increase with age, and almost 100% of the adult population suffers from dental caries since tooth decay keeps progressing throughout life [3]. The mean DMFT is around 5 almost all over the world, and a DMFT less than 5 is generally considered as low in the adult population [2]. That is why it is important to consider the dietary items that affect the progress of dental caries in adults if DMFT progresses beyond 5. This study has given the insight that this FFQ, which was translated into the local language (Urdu), may be implemented in a larger population, and in countries where Urdu is spoken and understood easily. Moreover, since the Cronbach’s alpha value (0.80) was almost similar to what was reported when used in the Japanese population [20], this FFQ may apply to any part of the world in any language. This will assist in the further exploration of the food patterns which may be specific for specific regions.

The findings of the present study not only confirmed that the DMFT level prevails as identified previously one and a half decades ago but also highlights the important point that the cereals which were more commonly consumed in industrialized countries are gaining in trend in lower-to-middle income countries such as Pakistan. The consumption of cereals has been attributed to the progressive prosperity of the middle-income population [35]. In addition, socioeconomic status is associated with the prevalence of caries among the adult population; however, this factor was not evaluated in the present study. Furthermore, in the present study, only cavitated lesions were taken into account, as well as lesions with activity in ICDAS (International Caries Detection and Assessment System) code greater than 2, and no non-cavitated or white spot lesions were considered carious. This may have influenced the outcomes of disease prevalence which were not reported in the study. Therefore, to explore dietary patterns with dental caries, multicenter investigations assessing relationship between dietary patterns, socioeconomic status, and systemic conditions and factors such as anemia, anthropometric measures such as BMI, diabetes, and other non-communicable diseases in the adult population are warranted.

## 5. Conclusions

This study suggests that higher dental caries levels with a DMFT equal to or greater than 5 in adults may be attributed to a higher intake of cereals.

## Figures and Tables

**Table 1:** Detailed description of study participants (*n* = 1730).

Variables			Mean ± SD/N (Percentage)
Age			32.65 ± 10.49
Gender		Male	796 (46.12)
		Female	934 (54.37)
Marital status		Single	779 (45.43)
		Married	951 (52.08)
BMI (kg/m^2^)		<18	467 (27.77)
		18–22.9	916 (53.50)
		23–<25	260 (15.20)
		≥25	87 (5.46)
No. of children			2 ± 1.83
Frequency of brushing	Once daily		495 (28.61)
Twice daily			1165 (67.34)
No brush			70 (4.04)
Dental visits	6 months		240 (13.87)
12 months			1389 (80.28)
>24 months			101 (5.83)
Fluoride level in toothpastes (as claimed by the manufacturer)		<1100 ppm	498 (28.78)
		≥1100 ppm	1232 (71.21)
Mean DMFT			5.74 ± 1.86
Mean D			3.29 ± 1.09
Mean F			0.76 ± 0.56
Mean M			1.67 ± 1.47

**Table 2:** Factor-loading matrix for four dietary factors (*n* = 1730).

Food Items	Food Patterns			
	Sweet Treat	Tea Meal	Cereal	Dairy and Nut
Candies (all kinds)	0.7153	0.1789	−0.1170	−0.0708
Ice cream or sherbet *	0.5628	−0.0471	0.0792	−0.0510
Bread filled with jam/fruit jam	−0.0468	0.8515	0.0733	−0.0397
Jam or jelly	0.2364	0.8179	0.1217	−0.0367
Sugared cereal	0.0102	0.1907	0.7211	0.1112
Plain cereal	0.0092	−0.0286	0.6416	0.1587
Dried fruit	0.0449	−0.0843	0.0785	0.6154
Plain yogurt	0.0785	−0.1501	0.0824	0.5812
Cold drinks	0.5049	0.0038	−0.1480	−0.0525
Cookies or biscuits	0.4897	0.1028	0.3547	0.0949
Cakes, donuts, or pies	0.3678	−0.1729	0.1916	0.2512
Bread	−0.0660	0.4732	0.0598	0.3916
Sugared yogurt	−0.1064	0.0034	−0.0251	0.5295
Rice	0.2271	−0.0967	0.1623	0.0822
Cheese	0.4555	0.0992	−0.0385	0.0726
Fresh fruit with sugar	0.1954	0.0630	0.4156	0.0840
Banana	−0.0684	0.0901	0.1523	0.4869
Milk	−0.1010	0.1354	−0.2002	0.1446
Fruit juice	0.3753	0.1231	−0.0055	0.0401
Sugar/honey in coffee/tea	0.1293	0.2069	0.0647	0.0606
Pudding or custard	−0.0381	0.2205	0.4144	−0.0663
Chocolates	−0.0381	0.2205	0.4144	−0.0663
Chips	0.2763	−0.3143	−0.5476	0.2776
Popcorn	−0.0272	0.2020	−0.0454	0.3237
Mithai (sweetmeat)	0.3505	−0.0041	0.0622	0.2026

Extraction method: principal component analysis, varimax rotation. * Pakistani sugary drink.

**Table 3:** Odds ratios and 95% confidence intervals for variables predicting ≥ 5 DMFT in adults.

Variables	Prevalence (%)	Crude OR (95% CI)	*p*-Value	Adjusted OR (95% CI) ǂ	*p*-Value
Age	--	1.06 (1.02–1.09)	0.001 *	1.06 (0.997–1.146)	0.060
Gender					
Male	279/796 (35%)	Reference		Reference	
Female	607/934 (65%)	3.42 (1.48–7.89)	0.004 *	2.81 (0.968–8.170)	0.057
Marital Status					
Single	296/779 (38%)	Reference		Reference	
Married	589/951 (62%)	2.86 (1.25–6.53)	0.013 *	0.52 (0.0977–2.781)	0.446
BMI	--	1.01 (0.92–1.11)	0.764	1.0 (0.8–1.12)	0.65
Frequency of Brushing					
Once daily	495 (28)	Reference		Reference	
Twice daily	1165 (67)	0.3 (0.2–1.9)	0.81	0.2 (0.1–2.1)	0.71
No Brushing	70 (5)	1.1 (0.2–1.2)	0.62	1.2 (0.1–1.5)	0.51
Dental Visits					
6 months	240 (13)	Reference		Reference	
12 months	1389 (80)	0.1 (0.1–1.7)	0.56	0.1 (0.1–1.5)	0.52
>24 months	101 (7)	0.02 (0.01–1.1)	0.73	0.01 (0.01–1.3)	0.7
Fluoride in Toothpastes †					
<1100 PPM	219/498 (44%)	Reference		Reference	
≥1100 PPM	677/1232 (55%)	1.54 (0.60–3.93)	0.361	1.55 (0.61–3.89)	0.25
Sweet Treat					
1st tertile	317/577(55%)	Reference		Reference	
2nd tertile	305/577 (53%)	0.85 (0.28–2.54)	0.78	0.83 (0.28–2.34)	0.68
3rd tertile	207/576 (36%)	0.53 (0.17–1.61)	0.26	0.55 (0.16–1.78)	0.25
Tea Meal					
1st tertile	236/577 (41%)	Reference		Reference	
2nd tertile	317/577 (55%)	1.59 (0.53–4.75)	0.406	1.60 (0.52–0.48)	0.39
3rd tertile	259/576 (45%)	1.16 (0.39–3.49)	0.780	1.18 (0.33–3.44)	0.66
Cereals					
1st tertile	155/577 (27%)	Reference		Reference	
2nd tertile	225/577 (39%)	1.69 (0.52–5.48)	0.377	1.17 (0.322–4.310)	0.803
3rd tertile	437/576 (76%)	9.04 (2.57–31.84)	0.001 *	4.36 (1.024–18.599)	0.046 *
Dairy and Nuts					
1st tertile	259/577 (45%)	Reference		Reference	
2nd tertile	305/577 (53%)	1.36 (0.45–4.05)	0.579	1.33 (0.41–3.39)	0.55
3rd tertile	241/576 (42%)	0.85 (0.28–2.55)	0.780	0.83 (0.28–2.33)	0.61

ǂ Adjusted for participants’ age, gender, marital status, and cereal consumption (dietary factor 3). † This level is that claimed by the toothpaste manufacturing company. * Significant *p*-value ≤ 0.05.

## Data Availability

Data of the study is available on contact form the corresponding author.
